# Esophagus involvement in systemic sclerosis: ultrasound parameters and association with clinical manifestations

**DOI:** 10.1186/s13075-021-02505-y

**Published:** 2021-04-21

**Authors:** Li Ma, Qingli Zhu, Yan Zhang, Jianchu Li, Yuxin Jiang, Dong Xu, Xiaofeng Zeng, Yong Hou, He Liu

**Affiliations:** 1 Department of Ultrasound, Peking Union Medical College Hospital, Chinese Academy of Medical Sciences, 1 Shuaifuyuan Street, Dongcheng District, Beijing, 100730 China; 2 Department of Radiology, Peking Union Medical College Hospital, Chinese Academy of Medical Sciences, 1 Shuaifuyuan Street, Dongcheng District, Beijing, 100730 China; 3 Department of Rheumatology and Clinical Immunology, Peking Union Medical College Hospital, Chinese Academy of Medical Sciences, National Clinical Research Center for Dermatologic and Immunologic Diseases (NCRC-DID), 1 Shuaifuyuan Street, Dongcheng District, Beijing, 100730 China

**Keywords:** Systemic sclerosis, Esophagus involvement, Ultrasound

## Abstract

**Background:**

The esophagus involvement in systemic sclerosis (SSc) is very common yet underestimated due to the lack of suitable screening tools. This study aims to explore the usefulness of ultrasound (US) in the assessment of esophagus involvement and to identify its relationship with clinical and CT manifestations.

**Methods:**

We performed transabdominal esophageal US in 38 SSc patients and 38 controls. US parameters including the abdominal esophagus length, esophagus wall thickness, shear-wave elastography, gastro-esophageal (His) angle, and reflux were compared. Relationships between distinguishable US parameters and clinical/CT parameters, such as gastro-esophageal reflux disease questionnaire (GERDQ), modified Rodnan skin score (mRSS), interstitial lung disease (ILD) score, the largest esophagus diameter (Dmax), and esophagus dilation percentage (%Eop), were evaluated.

**Results:**

Abdominal esophagus length was shorter in the SSc group than the control group (2.69 cm vs 3.06 cm, *P* = 0.018), whereas His angle and the angle change before and after drinking water were larger in the SSc group than the control group (121° vs 108°, *P <* 0.001; 7.97° vs 2.92°, *P* = 0.025). Reflux was more frequently seen in the SSc group than the control group (7/38 vs 0/38; *P* = 0.017). As for correlation with clinical and CT parameters, His angle was higher in patients with GERDQ ≥ 8 than GERDQ < 8 (116.5° vs 125.6°, *P* = 0.035). Patients with reflux showed higher ILD score than patients without (15.8 vs 9.6, *P* = 0.043). Furthermore, abdominal esophagus length was negatively correlated with %Eop and Dmax (*r* = − 0.573, *P* < 0.001; *r* = − 0.476, *P* = 0.003).

**Conclusion:**

US parameters of the esophagus can distinguish SSc patients from controls, as well as have correlations with clinical and CT characteristics. Our pilot study first shows that US can be used as a noninvasive and convenient method to evaluate the esophagus involvement in SSc.

## Introduction

Systemic sclerosis (SSc) is a rare rheumatic disease characterized by fibroproliferative alterations in the microvasculature, leading to fibrosis in the skin and internal organs [1, 2]. The esophagus is the most commonly affected organ in SSc, as reported to have occurred in 75–90% of SSc patients [1, 3, 4]. Esophagus dysfunction is associated with severe malabsorption, depression, and lower quality of life [5–7].

The esophagus involvement in SSc has been underestimated over a long time period. Up to date, the evaluation of the esophagus involvement is still mostly symptom-based, i.e., gastroesophageal reflux disease (GERD) symptoms. However, it is reported that up to 50% of SSc patients with esophagus involvement are asymptomatic or without typical symptoms [4]. The delayed diagnosis may lead to delayed treatments and irreversible damage to the esophagus [8, 9]. Other useful diagnostic modalities, such as 24-h pH monitoring, barium esophagography, esophageal manometry, upper GI endoscopy, and chest CT, cannot be used widely for routine clinical screening due to their invasiveness, radiation, complex procedure, and high expense [10, 11]. Therefore, a convenient screening tool for esophagus dysfunction is still lacking [12].

Transabdominal ultrasound (US) is a non-invasive, feasible tool that can evaluate the gastro-esophageal junction area and the lower esophageal sphincter (LES). Comparing to other optional studies that have unique strengths in evaluating specific aspects (e.g., 24-h pH monitoring measures esophagus pH, barium esophagography looks for strictures, esophageal manometry tests dysmotility, upper GI endoscopy looks at the mucosa, and chest CT looks for esophageal dilation), US can observe the morphology and texture of the esophagus wall, as well as the esophageal dynamics, such as gastro-esophageal angle (His angle) and its change, and reflux [13]. The visualization of the anatomical characteristics of the distal part of esophagus may reflect the underlying pathological vasculopathy and fibrosis, thus providing a unique angle for understanding GERD [3]. There have been several published studies confirming the value of the US in evaluating GERD in infants and children [14]. US can reach an 80–90% accuracy in diagnosing GERD as compared to 24-h pH monitoring [15, 16]. The new generations of US machines provide a high resolution in deeper organs, as well as offer shear-wave elastography (SWE) technique to quantify organ hardness, enabling the evaluation of adults’ esophagus. To our knowledge, the usefulness of US on the evaluation of esophagus involvement in SSc patients has not been reported previously.

Here, we designed a pilot study to validate the US parameters in the evaluation of esophagus involvement and explored their associations with clinical manifestations.

## Materials and methods

### Study participants

Thirty-eight patients and 38 controls participated in this cross-sectional study. For the patient group, subjects must fulfill the American College of Rheumatology (ACR)/ European League Against Rheumatism (EULAR) 2013 criteria for SSc, and only subjects with diffuse cutaneous systemic sclerosis (dcSSc) or limited cutaneous systemic sclerosis (lcSSc) were included [17, 18]. All patients were consecutively recruited at the Rheumatology Department of Peking Union Medical College Hospital between September 2018 and October 2019. For the control group, volunteers with no known underlying connective tissue diseases (CTD) or vascular diseases, including Raynaud’s phenomenon, diabetes, and hypertension, nor the upper gastrointestinal symptoms, were recruited. The GERD symptoms were assessed by the GERD questionnaire (GERDQ) as previously described [19]. A GERDQ ≥ 8 is considered to have GERD. Patients and controls were matched by age (less than 3 years’ difference) and sex. The study was approved by the ethics committee of Peking Union Medical College Hospital and followed institutional guidelines. All participants gave written informed consent, and the body photo used in this article was consented by one volunteer (Z.M.).

### Clinical and serological assessment

At entry, all patients with SSc underwent clinical and serological assessment. Data were collected including the patient’s age, gender, and disease duration. Disease duration was defined as the time between the first non-Raynaud symptom attributed to SSc and the date of US examination. The modified Rodnan skin score (mRSS) was determined by evaluating 17 skin sites in each patient, including the face, upper arms, forearms, dorsum of the hands, fingers, chest, abdomen, thighs, lower legs, and feet as previously described [20]. The symptoms of GERD were assessed by GERDQ as described above. The mRSS score and GERDQ were evaluated by an experienced physician specialized in rheumatology, who was trained at the EULAR Scleroderma Trials and Research group course [21]. The serological markers, such as antinuclear antibody (ANA), anti-centromere antibody (ACA), and Scl-70, were tested in the high-level rheumatological laboratory and recorded. The pulmonary function was performed for every patient, with forced vital capacity (FVC) and carbon monoxide diffusing capacity, single breath (DLCO, SB) recorded. All patients underwent the echocardiogram. Systolic pulmonary arterial pressure (PASP) pressure was measured by echocardiography through estimating right atrial pressure and measuring tricuspid regurgitation velocity. Pulmonary hypertension was defined as the PASP > 40 mmHg. None of participants in our study used promotility agents. Twenty patients were taking GERD medications (i.e. PPIs, antiacids) as needed. These patients were asked to stop taking GERD medications at least 24 h prior to US examinations.

### Ultrasound examinations

Participants were asked to fast for at least 8 h before US examination. The examinations were conducted by one radiologist (L.H.) who had experience with > 100 gastric US examinations using a SuperSonic Aixplorer machine (SuperSonic Imaging, SA, France) with the convex (C5–2) transducer. The radiologist was blinded to the diagnosis, clinical symptoms, relevant laboratory data, and any imaging or endoscopy results. Since the appearance of SSc patients was distinct from controls, it was hard to keep total blinding when performing ultrasound examinations. However, blinding was guaranteed at the measurements and analysis steps, since they were taken later without the presence of patients.

**Fig. 1:**
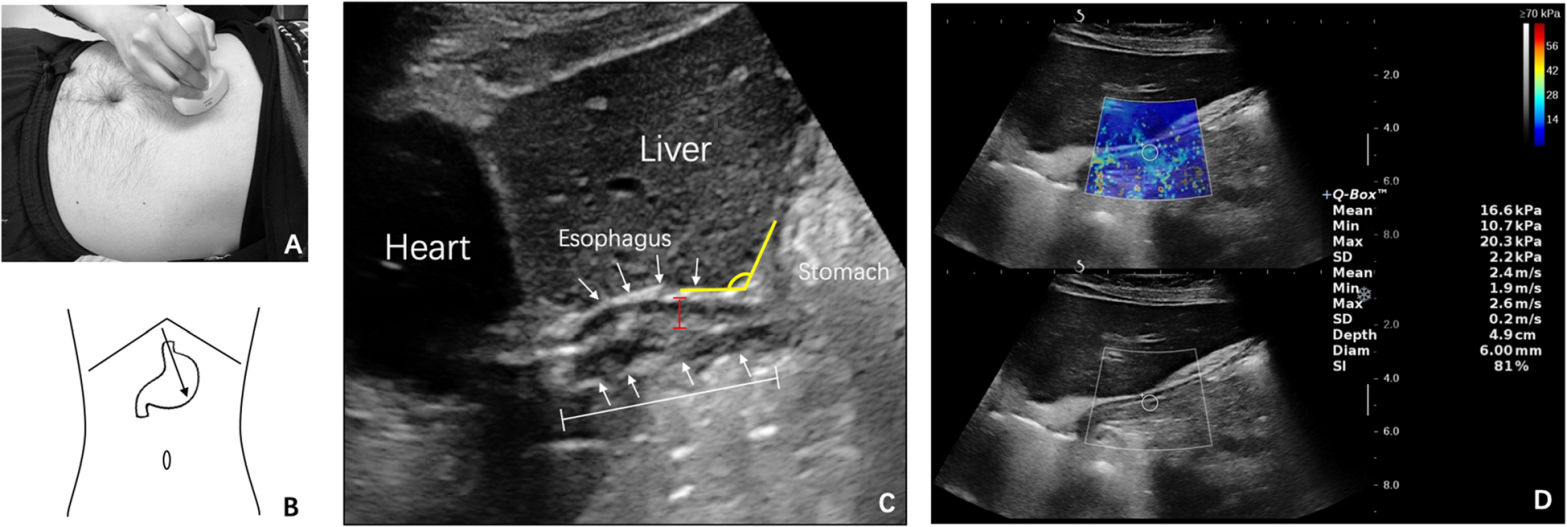
The measurements of US parameters. **a**, **b** The transducer was placed a little left-leaning under the xiphoid, to reveal the longitudinal abdominal esophagus and gastro-esophageal junction. **c** The US image showing the abdominal esophagus and gastro-esophageal junction. The esophagus is delineated as a linear structure (*arrows*) with the hypoechogenic wall and hyperechogenic lumen. The measurements of abdominal esophagus length (*white lines*), esophageal wall thickness (*red lines*), and His angle (*yellow lines*) are illustrated. C. SWE was obtained by placing the SWE box (*trapezoid*) on the abdominal esophagus, and the image was frozen after the colored signal was stable. A Q-box region of interest (*circle*) was positioned within the anterior wall of the abdominal esophagus. The mean elastic modulus within the Q-box was shown on the screen. Each measurement was performed three times. His angle, the gastro-esophageal angle; SWE, shear-wave elastography

The abdominal part of the esophagus and the gastro-esophageal junction was examined as previously described (Fig. 1) [22]. In short, the transducer was placed sagittally in the midline or a little left-leaning under the xiphoid, to reveal the abdominal esophagus longitudinally through the window of the left lobe of the liver. The SWE image was obtained by placing the SWE box on the abdominal esophagus, and the image was frozen after the colored signal was stable. Frozen photos, SWE images, and dynamic videos were recorded. Then the participants were asked to intake 300 ml of warm water. Videos were recorded right after water-drinking for at least 5 min. Frozen photos and SWE images were also saved after 5-min recording [23].

US parameters were chosen primarily based on their reported association with GERD. The length of the abdominal esophagus was measured from the point at which the esophagus traversed the diaphragm to the gastro-esophageal junction, which was identified on sonograms by a small triangular pad of gastric folds radiating from the cardia [14]. The esophageal wall thickness was measured on the anterior wall at the midpoint of the abdominal esophagus [22]. The His angle was delimited by the tangent line passed from the left fornix of the stomach and the long axis of the abdominal esophagus [24]. SWE measurements were referred to its mature application on other organs [25]. A round “Q-box” region of interest was positioned inside the SWE image and within the anterior wall of the abdominal esophagus. Then the mean elastic modulus within the “Q-box” was shown on the screen. Each measurement was performed three times, and the mean value was used for further analysis. Measurements of the length of the abdominal esophagus, the esophageal wall thickness, SWE, and His angle were performed both before and after water intake whenever the esophagus status is stable (no reflux or peristalsis). Reflux was carefully observed immediately after water intake at the gastro-esophageal junction.

To test the intra-observer reproducibility of the measurements, the same radiologist (L.H.) reviewed saved videos 1 month later and repeated all measurements. To assess interobserver reproducibility, another independent radiologist (M.L.), who had experience with > 50 gastric US examinations, reviewed saved videos and repeated all measurements.

### CT

The chest CT was performed on all SSc patients. Images were obtained using a C-100 scanner (GE Imatron, San Francisco, CA, USA) with a section thickness of 5 mm at full inspiration. For the esophagus, the images were set to the mediastinal window (window width 396 HU, window level 44 HU). The largest linear measurement of the esophageal air column was recorded as the largest esophagus diameter (Dmax) [26]. The number of sections with the esophagus air column more than 10 mm was divided by the total number of the esophageal sections to give a percentage of the open or dilated esophagus over the whole esophagus, namely esophagus dilation percentage (%Eop) [27]. A fluid level was recorded if there was an air-fluid level at any place in the esophagus. The interstitial lung diseases (ILD) score was evaluated as previously described [27]. In short, the CT images were set to lung windows (window width 1465 Hounsfield Units (HU), window level − 498 HU). Lungs were divided into 6 zones, and each zone was scored from 0 to 3 by severity in each of the following categories: ground glass, fibrosis (reticular or scarring), honey-combing, and consolidation. The esophagus parameters and ILD score were assessed by an experienced radiologist who was blinded to clinical and laboratory data.

### Statistics

We performed statistical analysis using SPSS software version 23 (IBM Corp., Armonk, NY, USA). Descriptive statistics were reported as number (percent) for categorical variables, or median (range) for continuous variables. Intra-observer and inter-observer reproducibility were evaluated using intraclass correlation coefficient (ICC) analysis which was graded as follows: poor (< 0.20), fair (0.20 to 0.40), moderate (0.40 to 0.60), good (0.60 to 0.80), or very good (0.80–1.00). The independent t-test was used to compare continuous variables and categorical variables were compared with Pearson’s chi-square test or Mann-Whitney *U* test. Correlations between variables were analyzed using Pearson’s correlation for two variables with normal distribution, or Spearman’s correlation for two variables with non-normal distribution. A *P* value < 0.05 was considered statistically significant. An experienced biomedical statistician reviewed the statistical methods used in this study.

## Results

### Patient characteristics

Thirty-eight SSc patients and 38 age- and sex-matched controls were recruited in the study. The baseline characteristics of all participants are shown in Table 1. There were 8% (3/38) males in both groups. The average age was 51.3 ± 12.6 in the SSc group and 52.2 ± 12.9 in the control group, with no statistical difference (*P* = 0.76). A total of 21 SSc patients were diagnosed as GERD with a GERDQ ≥ 8, and 17 patients were not diagnosed GERD with a GERDQ < 8. No one in the control group had GERD. For SSc patients, the average disease duration was 11.2 ± 6.6 years. The mRSS score was 4.6 ± 3.9. The FVC was averagely 87.8% of predicted, and DLCO was averagely 63.7% of predicted. In all, 16 (42%) patients were positive for Scl-70, 33 (87%) were positive for ANA, and 7(18%) were positive for ACA. No patients had pulmonary hypertension.

**Table 1:** Baseline characteristics of enrolled participants

	Patient (***n*** = 38)	Control (n = 38)	***P***
**Male,** ***n*** **(%)**	3 (8%)	3 (8%)	1
**Age (years), mean ± SD**	51.3 ± 12.6	52.2 ± 12.9	0.76
**SSc characteristics**			
**Disease duration(years), mean ± SD**	11.2 ± 6.6		
**mRSS, mean ± SD**	4.6 ± 3.9		
**FVC (actual/predicted, %), mean ± SD**	87.8 ± 17.2		
**DLCO SB (%), mean ± SD**	63.7 ± 13.7		
**Scl-70,** ***n*** **(%)**	16 (42%)		
**ANA,** ***n*** **(%)**	33 (87%)		
**ACA,** ***n*** **(%)**	7(18%)		
**Pulmonary hypertension,** ***n*** **(%)**	0		
**GERDQ (< 8:≥ 8)**	17:21	38:0	

*Abbreviations*: *SD* standard deviation, *SSc* systemic sclerosis, *MRSS* the modified Rodnan skin score, *GERDQ* gastro-esophageal reflux disease questionnaire, *ANA* antinuclear antibody, *ACA* anti-centromere antibody, *DLCO SB* carbon monoxide diffusing capacity, single breath, *FVC* forced vital capacity

### Comparison of ultrasound parameters between SSc and control groups

The differences of US parameters between the SSc and control groups are shown in Table 2. The abdominal esophagus length was shorter in the SSc group than the control group (before drinking water, 2.69 cm vs 3.06 cm, *P* = 0.018; after drinking water, 2.64 cm vs 3.03 cm, *P* = 0.016), with no significant change before and after drinking water. His angle was larger in the SSc group than in the control group both before and after drinking water (before drinking water, 121° vs 108°, *P <* 0.001; after drinking water, 129° vs 111°, *P <* 0.001). Also, the angle change was larger in the SSc group (7.97° vs 2.92°, *P* = 0.025). There were 7 out of 38 patients who showed reflux after drinking water, and no one in the control group showed reflux (*P* = 0.017). There were no differences in esophagus wall thickness (*P* = 0.890 and 0.185, before and after drinking water) and SWE (*P* = 0.703 and 0.416, before and after drinking water) between the two groups, both before and after drinking water.

**Table 2:** Comparison of ultrasound parameters between SSc and control group

	SSc group	Control group	***P***
Abdominal esophagus length (cm)^*^			
**Before drinking water**	2.69	3.06	0.018
**After drinking water**	2.64	3.03	0.016
Esophagus wall thickness (mm)^*^			
**Before drinking water**	3.47	3.49	0.890
**After drinking water**	3.66	3.91	0.185
**His angle (°)**			
**Before drinking water**	121	108	< 0.001
**After drinking water**	129	111	< 0.001
**Angle change**	7.97	2.92	0.025
SWE (kPa)^*^			
**Before drinking water**	5.52	6.20	0.703
**After drinking water**	4.58	3.56	0.416
**reflux**	7/38	0/38	0.017

^*^There is no significant difference before and after drinking

*Abbreviations*: *SSc* systemic sclerosis, *His angle* the gastro-esophageal angle, *SWE* shear-wave elastrography

These results suggest that abdominal esophagus length, His angle, and reflux are characteristic parameters in SSc patients, whereas esophagus wall thickness and SWE had no significant differences between patients and controls in our study.

### Intra-observer and inter-observer reproducibility

For intra-observer reproducibility, the ICC of abdominal esophagus length, esophagus wall thickness, His angle, SWE and reflux was 0.760 (95% confidence interval [CI]: 0.646–0.841, good), 0.206 (95% CI: − 0.019–0.411, fair), 0.883 (95% CI: 0.822–0.924, very good), 0.352 (95% CI: − 0.157–0.713, fair), and 1 (very good). For inter-observer reproducibility, the ICC of abdominal esophagus length, esophagus wall thickness, His angle, and reflux were 0.818 (95% CI: 0.728–0.881, very good), 0.803 (95% CI: 0.705–0.870, very good), and 0.613 (95% CI: 0.368–0.778, good) (Table 3).

**Table 3:** Intra-observer and inter-observer reproducibility

	Intra-observer reproducibility	Inter-observer reproducibility
	ICC (95% CI)	ICC (95% CI)
**Abdominal esophagus length**	0.760 (0.646–0.841)	0.818 (0.728–0.881)
**Esophagus wall thickness**	0.206 (− 0.019–0.411)	/
**His angle**	0.883 (0.822–0.924)	0.803 (0.705–0.870)
**SWE**	0.352 (− 0.157–0.713)	/
**Reflux**	1	0.613 (0.368–0.778)

*Abbreviations*: *ICC* intraclass correlation coefficient, *CI* confidence interval, *His angle* the gastro-esophageal angle, *SWE* shear-wave elastography

These results indicate that abdominal esophagus length, His angle, and reflux had a good intra-observer and inter-observer reproducibility. SWE and esophagus wall thickness had poor intra-observer reproducibility.

### Correlation between ultrasound parameters and clinical/CT manifestations in SSc patients

**Fig. 2:**
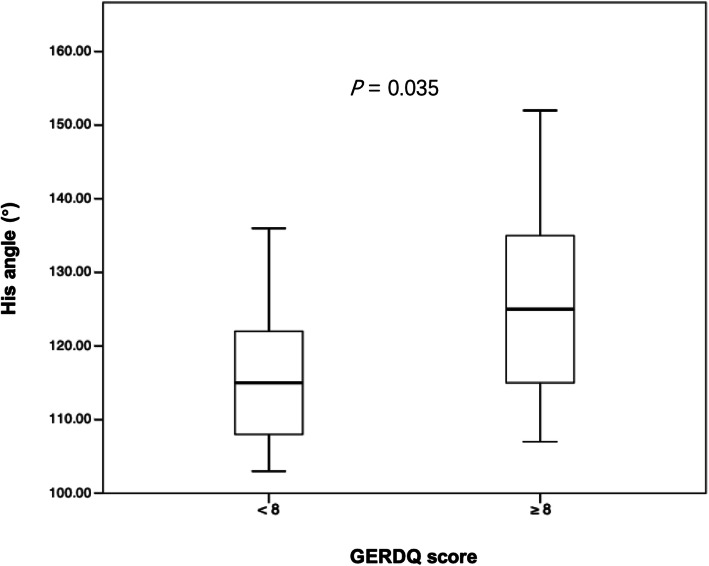
His angle in SSc patients with GERDQ < 8 and GERDQ ≥ 8. Patients with GERDQ < 8 has a smaller His angle than patients with GERDQ ≥ 8, with a *P* value < 0.05. GERDQ, gastro-esophageal reflux disease questionnaire; His angle, the gastro-esophageal angle

**Fig. 3:**
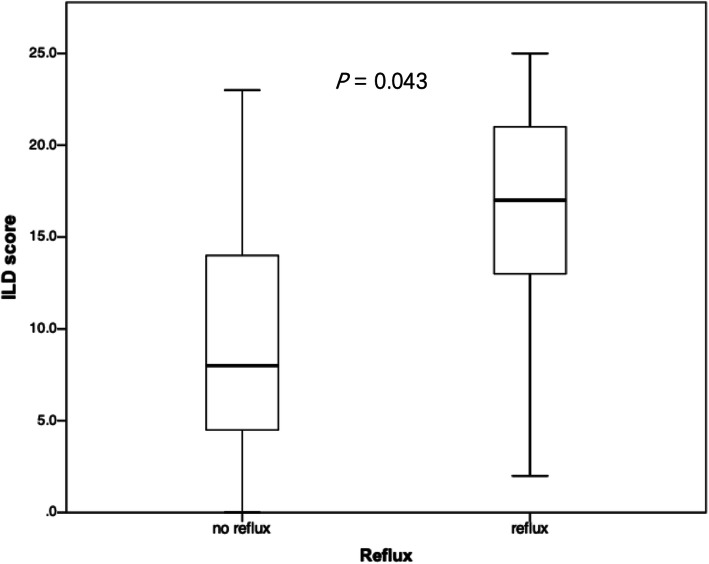
ILD score in SSc patients with/without reflux on US. ILD score is higher in patients with reflux than without reflux, with a *P* value < 0.05. ILD, interstitial lung disease

**Fig. 4:**
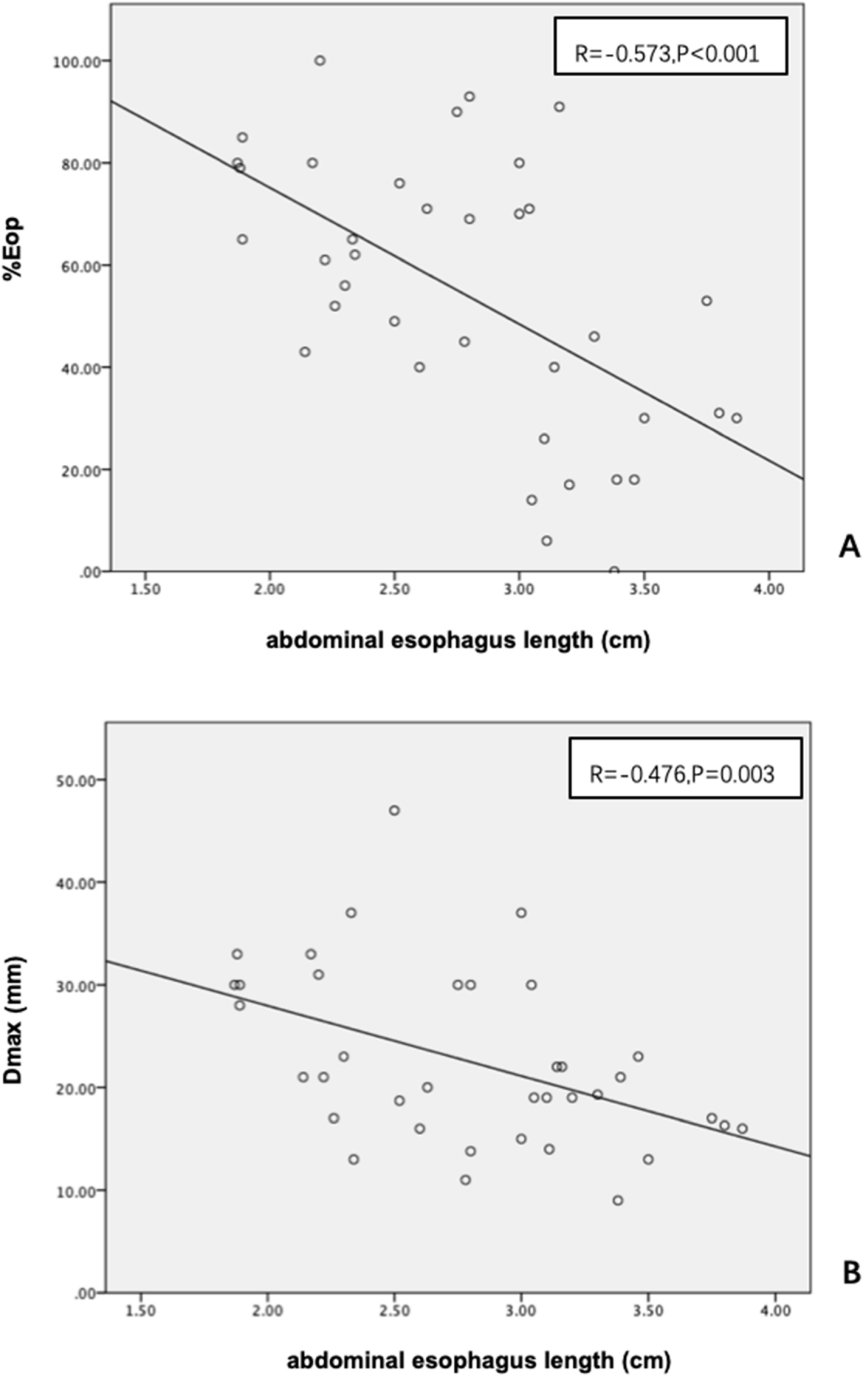
The correlations between abdominal esophagus length and CT parameters. **a** Abdominal esophagus length is negatively correlated to the esophagus dilation percentage (%Eop). **b** Abdominal esophagus length is negatively correlated to the largest esophagus diameter (Dmax)

The correlation between ultrasound parameters (abdominal esophagus length, His angle, and reflux) with clinical factors (mRSS, GERDQ) and CT parameters (Dmax, %Eop, ILD score) was analyzed. We found that His angle was higher in SSc patients with GERD (GERDQ score ≥ 8) than without (GERDQ score < 8) (116.5°vs 125.6°, *P* = 0.035, Fig. 2). Abdominal esophagus length and reflux did not show a significant difference in patients with GERD or without (*P* = 0.154 and *P* = 0.271, respectively). The ILD score was higher in SSc patients with reflux on ultrasound than without (15.8 vs 9.6, *P* = 0.043, Fig. 3). The abdominal esophagus length was negatively correlated with %Eop (*r* = − 0.573, *P* < 0.001) and Dmax (*r* = − 0.476, *P* = 0.003) on CT (Fig. 4). None of the US parameters were correlated with mRSS. Three patients have records of endoscopy. Two patients had reflux observed on US, and both of them had reflux esophagitis under endoscopy. Correspondingly, on the other patient, no reflux was observed, while there was no esophagitis under the endoscopy.

These results indicate that US parameters were associated with clinical and CT manifestations in SSc patients.

## Discussion

As the esophagus is a commonly involved organ of SSc, a practical and convenient screening tool to objectively evaluate the existence and severity of esophagus involvement is still lacking. Transabdominal US provides a non-invasive method to picture precisely the gastro-esophageal junction, the most important portion for esophageal dysfunction. The novelty of our study is that we first reported a spectrum of US parameters that can be used to detect the esophagus involvement in SSc patients, and that these new parameters correlated to SSc clinical and CT markers.

The US parameters we adopted in this study are summarized from literature mostly reporting the US evaluation of GERD in infants and children [14].

In the literature, the abdominal esophagus length is a commonly used parameter, that is shorter in GERD patients as compared to normal controls in both our and previous studies [2, 22, 28, 29]. According to the in vitro experiments by De Meester et al., adequate length of intraabdominal esophagus is the key determinant of maintaining the competency of the cardia [28]. The underlying reason of the shortage of the abdominal esophagus length can be congenital, but in SSc patients may also be due to the fibrosis and contracture of the esophagus wall. The normal range of the abdominal esophagus length in adults is not reported before, since most studies focused on children and infants. However, De Meester et al. reported a length < 2 cm has been highly associated with GERD in adults [28].

The observation of the reflux flow right after water intake by US is the most direct evidence of gastro-esophageal reflux. It has been reported that the reflux detected by US has a high sensitivity (95.5%) and a positive predictive value (83.3%) with reference to pH monitoring in children [15, 30]. In our study, we found reflux a discriminating parameter between SSc and control group, yet the sensitivity is to be determined.

His angle is an important indicator of the sliding hiatus hernia in children. Previous studies showed that His angle is a stable, age-independent parameter, which is larger in GERD patients than controls [24, 31, 32]. Halkeiwicz et al. reported a normal range of 70–100° [24]. We firstly applied His angle on SSc patients and found that it was larger in SSc patients than controls. Moreover, the angle change before and after water intake was larger in SSc patients than in controls. This can be explained by that the stiffness of the esophagus and stomach wall in SSc decreases the elasticity, making it harder to maintain the original form under pressure, and reflected as a bigger angle change.

The esophagitis caused by gastric acid regurgitation, along with the excessive deposition of collagen fibers caused by SSc, results in the thickness of the esophageal wall [11, 29]. Literature has reported that the esophageal wall is thicker in GERD patients than healthy controls [29, 31, 33]. Theoretically, SWE can provide quantitative measurements of the esophageal stiffness. We measured the esophageal wall thickness and SWE value before and after water drinking, with the intention to see the deformation of the abdominal esophagus. Unfortunately, both esophageal wall thickness and esophageal SWE failed to show good repeatability in our study. The range of the esophagus wall thickness is only 2–5 mm, thus it is easy to be affected by measurement errors. During our practice, SWE is highly affected by the heartbeat, breath, and the complication of the esophagus structure. Further research is needed to find a suitable way to use SWE.

In our study, US parameters had a fair-to-good correlation with CT parameters (Dmax, %Eop). No previous studies had reported the correlation between the two imaging modalities. Our study proved that, although evaluating esophagus from different perspectives, US and CT can be complementary tools to reflect esophagus condition in SSc. We also found that His angle has a positive correlation with GERDQ, an auxiliary method for GERD diagnosis. These results imply that US is able to evaluate the esophagus status in SSc patients.

Accumulating evidence has suggested that esophageal disease may be an independent contributor to ILD, a condition that is strongly associated with increased morbidity and mortality [8, 10, 34, 35]. Previous studies have shown a negative correlation between esophagus dilation and pulmonary function test [8, 10, 34]. Our study demonstrated that SSc patients with reflux on US tend to have higher ILD scores than patients with no reflux, confirming the relationship between esophagus dysfunction and ILD in SSc. We did not find correlations between US parameters and pulmonary function test indicators, possibly due to the small sample size. Several studies have shown that the extent of skin involvement is correlated with internal organ involvement, thereby we looked at the correlation between mRSS and US parameters [36, 37]. However, no association was found between US parameters and mRSS. Since the enrolled SSc patients were not naïve, the skin thickness is under the influence of the long-term medication, which disturbs the original condition of the skin.

There are several limitations to our study. First, we did not perform two independent examinations to test the intra- and inter-observer reliability for every participant, but one examination with individual measurements on photos and videos by the same or different radiologists. This is because the repetition of the examination required a period of time waiting for the stomach emptying, which was not quite ethical, as well as was hard to get consent when patients were fasted for 8 h, including some of whom were asked to stop GERD medication for 24 h. On the other hand, a pre-test in 3 healthy volunteers with complete individual examinations confirmed the results of the presented intra-observer reliability and inter-observer reliability. Second, we did not compare US parameters with classical examination methods for GERD, such as 24-h pH monitoring, barium esophagography, esophageal manometry, or upper GI endoscopy, due to their complexity and invasiveness leading to the limited use in China. Another obvious limitation is that the sample size is relatively small despite our promising findings, which leaves the validation of our preliminary results for future studies.

## Conclusion

In this pilot study, we found that transabdominal US parameters for the esophagus, such as abdominal esophagus length, His angle, and reflux, have significant differences between SSc patients and controls. Moreover, they are associated with CT parameters (i.e. the largest esophagus diameter, esophagus dilation percentage, ILD score), and GERDQ. Therefore, we conclude that US has the potential to be a pragmatic tool for detecting the esophagus involvement in SSc.

## Data Availability

The datasets during and/or analyzed during the current study are available from the corresponding author on reasonable request.
